# Dynamic and static calcium gradients inside large snail (*Helix aspersa*) neurones detected with calcium-sensitive microelectrodes

**DOI:** 10.1016/j.ceca.2006.07.010

**Published:** 2007-04

**Authors:** Roger C. Thomas, Marten Postma

**Affiliations:** Department of Physiology, Development and Neuroscience, University of Cambridge, Cambridge CB2 3EG, UK

**Keywords:** Neurone, Intracellular calcium, Diffusion

## Abstract

We have used quartz Ca^2+^-sensitive microelectrodes (CASMs) in large voltage-clamped snail neurones to investigate the inward spread of Ca^2+^ after a brief depolarisation. Both steady state and [Ca^2+^]_*i*_ transients changed with depth of penetration. When the CASM tip was within 20 μm of the far side of the cell the [Ca^2+^]_*i*_ transient time to peak was 4.4 ± 0.5 s, rising to 14.7 ± 0.7 s at a distance of 80 μm. We estimate that the Ca^2+^ transients travelled centripetally at an average speed of 6 μm^2^ s^−1^ and decreased in size by half over a distance of about 45 μm. Cyclopiazonic acid had little effect on the size and time to peak of Ca^2+^ transients but slowed their recovery significantly. This suggests that the endoplasmic reticulum curtails rather than reinforces the transients. Injecting the calcium buffer BAPTA made the Ca^2+^ transients more uniform in size and increased their times to peak and rates of recovery near the membrane. We have developed a computational model for the transients, which includes diffusion, uptake and Ca^2+^ extrusion. Good fits were obtained with a rather large apparent diffusion coefficient of about 90 ± 20 μm^2^ s^−1^.This may assist fast recovery by extrusion.

## 1 Introduction

Changes in free intracellular calcium ([Ca^2+^]_*i*_) play a vital role in intracellular signalling, notably in coupling electrical excitation to secretion, contraction and other activation processes [1,2]. Many of the processes activated by a rise in [Ca^2+^]_*i*_ are believed to occur in the nucleus [3,4], which in large neurones may be far from the cell membrane. It is well-established that a brief depolarization of most nerve cells causes an influx of Ca^2+^ ions across the plasma membrane and thus a local rise in [Ca^2+^]_*i*_. The spatiotemporal characteristics of this rise in calcium are defined by the interactions between Ca^2+^ ions and the cytoplasmic milieu. Calcium-induced calcium release (CICR) processes via either IP3 or ryanodine receptors can facilitate the regenerative spread of the calcium signal throughout the cell, whilst immobile calcium buffers will slow and attenuate the signal. If the increase in sub-membrane [Ca^2+^]_*i*_ is to act as a signal at a distance, the speed at which the Ca^2+^ ions travel from the cell membrane to their target depends critically on the rate of diffusion of Ca^2+^ ions in the cytoplasm. Most studies of calcium homeostasis and signalling in nerve and muscle cells have been made using optical methods to measure [Ca^2+^]_*i*_ via the binding of Ca^2+^ ions by fluorescent or other indicators [5]. Such indicators tend to increase intracellular Ca^2+^ buffering and mobility [6]. Many authors (for example [7–11]) have modelled the effect of mobile indicators on Ca^2+^ transients, but very few have produced data on intracellular Ca^2+^ movement from the periphery towards the centre inside real neurones in the absence of such alien additives. We now present such data, obtained with Ca^2+^-sensitive microelectrodes (CASMs), for the first time.

CASMs offer an alternative but rarely-used method for measuring [Ca^2+^]_*i*_. They have the advantage of measuring [Ca^2+^]_*i*_ in a small volume without adding any extraneous compounds which might interact with Ca^2+^ throughout the cell. But they are rather slow to respond, and only a realistic choice for large cells since they tend to create a leak at the site of penetration [12–15].

In previous work, with CASMs made from aluminosilicate glass, the CASMs were rarely moved once a stable potential had been obtained from deep inside the neurone [e.g. 13,14,16]. Recently a modified electrode construction technique has allowed us to use CASMs with very slender tips and longer lives made from thin-walled quartz glass. We have also found it possible, with a motorized micromanipulator, to move the CASMs in repeatable steps up and down, usually without apparently causing damage to the cell.

We have sought to record Ca^2+^ transients at different distances from the inside of the membrane at the far side of the penetrated cell. Our recordings at different depths were quite repeatable, and enabled us to reconstruct the radial inward movement of the entering Ca^2+^ towards the centre of the cell. We have done this in large neurones with initially no added Ca^2+^ indicator or buffer. Although our method does tend to induce a leak of Ca^2+^ ions at the penetration site, we believe that the leak simply increases the steady state level without apparently influencing the kinetics of the Ca^2+^ transients. We investigated the role of the endoplasmic reticulum by blocking its Ca^2+^ uptake pump with cyclopiazonic acid. We also injected the mobile Ca^2+^ buffer BAPTA, which has very similar Ca-buffering properties to many fluorescent Ca^2+^ indicators, to measure its effects on Ca^2+^ diffusion. We have modelled the Ca^2+^ increases mathematically and have been able to model the transients closely by assuming that calcium signalling in these cells can be described by Ca^2+^ influx, diffusion, extrusion, sequestration and buffering. Good fits were obtained with a diffusion coefficient of about 90 ± 20 μm^2^ s^−1^, which is higher than reported in other cell types.

## 2 Materials and methods

### 2.1 General

All experiments were done on large (150–250 μm diameter) snail neurones in isolated sub-oesophageal ganglia [14,16]. Cells were voltage-clamped using two microelectrodes to −50 mV, and depolarized at intervals by 40 or 50 mV to generate an influx of Ca^2+^ ions. The resulting changes in [Ca^2+^]_*i*_ were measured using CASMs. The main differences from previous methods were that we made the CASMs with quartz rather than aluminosilicate glass and used a motorized micromanipulator (Luigs & Neumann, Ratingen, Germany) to move the CASM up and down in reproducible steps. Rotating the Z-control wheel by 360° moved the CASM up or down by 40 μm, thus for a 20 μm step we rotated it manually by 180°.

### 2.2 Preparation

An aestivating snail, *Helix aspersa*, was killed humanely by rapid removal of the circumoesophageal ring of ganglia. These ganglia were mounted dorsal side uppermost on a plastic bath insert. The thick connective tissue covering the back of the visceral and right and left pallial ganglia was then removed and the bath insert with ganglia was mounted in the experimental chamber at an angle of 50° to the vertical. Once covered with snail Ringer (flow rate 1.0–1.2 ml min^−1^, bath volume 0.1 ml) the inner connective tissue was torn with a fine tungsten hook to expose some of the neurones. All experiments were carried out at room temperature, 18–22 °C, starting at least an hour after the dissection.

### 2.3 Solutions

The normal snail Ringer contained (mM): 80 NaCl, 4 KCl, 7 CaCl_2_, 5 MgCl_2_, 20 HEPES, titrated with NaOH to pH 7.5. Nominally Ca-free Ringers had the same composition but with no CaCl_2_ and 12 mM MgCl_2_. The EGTA Ca-free solution had an additional 1 mM EGTA. The CO_2_ Ringer was the same as normal Ringer except that it had 20 mM NaHCO_3_ instead of HEPES, was bubbled with 2.5% CO_2_ in air and contained 0.1 mM NaH_2_PO_4_. The high K low Ca solution for BAPTA iontophoresis calibration contained 100 KCl, 5 NaCl, 0.6 MgCl_2_, and 10 mM HEPES. The pH was adjusted to 7.2 with KOH. Cyclopiazonic acid (CPA) was kept frozen as a 50 mM stock solution in dimethyl sulphoxide and diluted in snail Ringer on the day of use. All these chemicals were from Sigma.

### 2.4 Microelectrodes

Conventional micropipettes were pulled from 1.2 mm filamented borosilicate glass tubing and backfilled with either 2 M CsCl (for passing current), or 2 M KCl (for recording membrane potential). Tips were broken if necessary by touching a pin in the bath to give resistances between 5 and 15 MΩ. BAPTA was iontophoresed from similar micropipettes filled with 100 mM BAPTA (K salt). CASMs were made by a method refined from those described previously [16,17]. Every few days we pulled 10–12 long, sharp micropipettes from unfilamented quartz tubing (1.0 mm o.d., 0.7 mm i.d.) using a Sutter P-2000 puller (Sutter Instrument Co, Novato, Cal.). We then broke the tips on a glass rod to o.d. 1.0–1.5 μm. We then left the micropipettes in air for 2–4 h before silanizing them with bis(dimethylamino)dimethylsilane (Fluka 14755) as described before [16].

Individual CASMs were prepared as follows on the day of the experiment. First we backfilled 2 or 3 silanized micropipettes with 1 mM CaCl_2_ (instead of the 100 mM KCl, 0.1 mM CaCl_2_ used before, which we realised shortened the CASM's useful life) using a 10 ml syringe connected to the back of each micropipette via a silicone rubber tube to force air out of the tips. We then sucked a column (150–250 μm) of sensor cocktail into the tip of each micropipette, taking care to avoid any air gap between cocktail and backfill. The cocktail composition was (mg/g): 12 Ca ionophore ETH 129 (Fluka 21193), 6 Na tetrakis[3,5-bis(trifluoromethyl)phenyl]borate, 200 2-nitrophenyloctylether, 17 high molecular weight PVC, and 765 tetrahydrofuran (all from Fluka). Filled microelectrodes were left in air to allow the tetrahydrofuran to evaporate for at least 2 h before use, and had useful lives of 2–3 days. During the experiment we applied 80–120 kPa of air pressure to the back of the CASM, since this maximizes its response [15]. Before use each microelectrode was tested in the experimental bath, initially perfused with normal snail Ringer. Once the potential had stabilised the perfusate was switched to Ca-free (EGTA) Ringer's solution. Any electrode showing a potential change of less than 150 mV was discarded. The potentials recorded from the CASMs during the experiments were displayed as *V*_Ca_ (*V*_m_ being automatically subtracted when both electrodes were in the same cell) and we have not converted them either to pCa or nM free [Ca^2+^]_*i*_ except for modelling purposes. Calibration of CASMs is difficult because, in our hands, their responses tend to change when they are withdrawn from a cell at the end of an experiment. Previous work [14] suggests that their responses are essentially linear with pCa between 2 and 7(10 mM–100 nM), with a potential change of 28 mV per decade. Half-response times ranged from 0.2 to 0.5 s, but were not routinely measured. Results were discarded if on withdrawal the electrode potentials had changed by more than 7 mV.

### 2.5 Estimating the BAPTA intophoresis transport number

We first used CASMs to measure how much BAPTA was required to change the *V*_Ca_ of 1 ml of the high K, low Ca solution from −50 to −110 mV (CASM zeroed in normal snail Ringer). The BAPTA was injected into the 1 ml aliquots using an oocyte microinjector. The average quantity of BAPTA needed was 75 nmol. We then made droplets (volumes 15–25 nl) of the same solution under oil, measured *V*_Ca_ as with a snail neurone, and injected BAPTA iontophoretically. We found that to achieve the equivalent *V*_Ca_ change as in the 1 ml samples we had to inject an average 0.6 μC nl^−1^, giving a transport number of 0.01.

### 2.6 Data collection and analysis

Potentials from the microelectrodes were led via Ag/AgCl wires to preamplifiers in the cage. Outputs from these and the clamp amplifier were filtered (8-pole Bessel) and recorded at 20 Hz on a PC via a CED micro 1401 interface and Spike 2 data collection program (Cambridge Electronic Design, UK). The CASM potential was differentially recorded with the *V*_m_ microelectrode potential as reference. Figures were prepared from the CED data after loading into Microsoft EXCEL. Large spikes in the *V*_Ca_ records generated by electronic pickup were erased, and the clamp current records were restricted in range. For more details of most of the apparatus see [14,16]. Data are presented as means ± S.E.M. of *n* observations. The statistical significance of observed shifts in means was determined by a one-tailed Wilcoxon rank sum test. The difference between means was considered significant when the *p*-value was higher than 0.95. For baseline adjustment, selecting transients, time to peak and exponential fitting, a home made Matlab script with a graphical user interface was used.

### 2.7 Computational modelling

There is an extensive body of literature on the modelling of Ca^2+^-signalling in a wide variety of cell types, for example [18–21]. In this study we set out to develop a minimal computational model of Ca^2+^-signalling in snail neurones. We used the model to validate and explain the results obtained from the experiments. Furthermore, we used the model to estimate the mobility of Ca^2+^ in these large neuronal cell bodies, which have not been exposed to mobile Ca^2+^-indicators.

The model includes the following dynamic processes: Ca^2+^ diffusion, influx, extrusion, sequestration and buffering. We assume that influx and extrusion mechanisms at the plasma membrane are uniformly distributed over the cell's surface. The CASM and the axon attached to the soma only represent a small fraction of the cell's volume (<1%) and surface area (<5%), furthermore transients were measured near the far side of the cell, opposite to the site of penetration. Since we could not detect any major deformation of the cell looking through the microscope we assume that the Ca^2+^ transients measured close to the membrane can be modelled with spherical symmetry. Using these assumptions we can reduce the three-dimensional diffusion problem to a one-dimensional radial diffusion equation in a sphere. Similar computational models for Ca^2+^ diffusion in a spherical cell can be found elsewhere [9,22].

The removal of Ca^2+^ after a brief influx is relatively slow, with time constants ranging from 5 to 15 s, while Ca^2+^ buffering occurs on the micro- and millisecond time scale. We therefore can separate these different processes kinetically and remove the explicit buffering terms from the transport equation [20]. In so doing we have to replace the Ca^2+^ diffusion coefficient *D*_Ca_ (μm^2^ s^−1^) with an apparent diffusion coefficient *D*_app:_

(1) $$D_{\text{app}}=\frac{D_{\text{Ca}}+D_{\text{e}}\kappa_{\text{e}}+D_{\text{b}}\kappa_{\text{b}}}{1+\kappa_{\text{e}}+\kappa_{\text{b}}}\text{,}$$

Here we used the notation from [7]; *κ*_e_ andκ_b_ denote the endogenous and exogenous Ca^2+^ binding ratio, respectively; the associated diffusion coefficients are *D*_e_ and *D*_b_. The diffusion coefficient of free cytoplasmic Ca^2+^ is denoted by *D*_Ca_ and was assumed to be 220 μm^2^ s^−1^ [23]. Furthermore, all other transport processes have to be scaled down by the total Ca^2+^ binding ratio *κ*_tot_ = 1 + *κ*_e_ + *κ*_b_.

The total Ca^2+^ binding ratio *κ*_tot_ depends on the Ca^2+^ concentration; however, when the concentration is much lower than the dissociation constant it may be approximated to be constant. Moreover, although some data are available on buffering in snail neurones [24], precise measurements that separate the buffering and sequestration processes are not. We have therefore worked with apparent fluxes and apparent parameters in most cases. Multiplication of these apparent parameters with the total buffering power should yield the real parameters.

The general transport equation that describes the calcium dynamics in the cell, taking into account the different aforementioned considerations then becomes:

(2) $$\frac{\partial {\left[\text{C}{\text{a}}^{2+}\right]}_{i}}{\partial t}=D_{\text{app}}\frac{1}{r^{2}}\frac{\partial }{\partial r}r^{2}\frac{\partial }{\partial r}{\left[\text{C}{\text{a}}^{2+}\right]}_{i}+r_{\text{sv}}j_{\text{basal},\text{app}}+r_{\text{sv}}j_{\text{in},\text{app}}+r_{\text{sv}}j_{\text{out},\text{app}}+J_{\text{seq},\text{app}}\text{,}$$

The first term on the right hand side represents the radial diffusional process that is driven by the concentration gradient in the cell. The transmembrane fluxes *j*_in_,_app_ and *j*_out_,_app_ describe the apparent influx of calcium through channels and apparent efflux of calcium by the pump, respectively, *J*_seq,app_ denotes the apparent calcium flux associated with sequestration to stores and mitochondria in the cell's cytoplasm and finally *j*_basal_,_app_ represents the basal influx of Ca^2+^ at rest. The surface-to-volume ratio *r*_sv_ (m^−1^) is necessary to convert the flux density *j* (mol m^−2^ s^−1^) into a concentration flux *J* (mM s^−1^). The concentration [Ca^2+^]_*i*_ and all fluxes are functions of position *r* (m) and time *t* (s); however, for readability we do not write this explicitly.

There are few precise quantitative data available about the different transport processes involved. To prevent the introduction of many unknown parameters we therefore kept the mechanistic complexity to a bare minimum. Consequently we modelled the influx as a square pulse with the same duration as the depolarisation:

(3a) $$j_{\text{in},\text{app}}=-I_{\text{Ca},\text{app}}\frac{1}{2FS}\text{,}$$

where the apparent Ca^2+^-current denoted by *I*_Ca,app_ (A) relates to the real current as follows: *I*_Ca,app_ = *I*_Ca_/*κ*_tot_. *S* denotes the surface area (m^2^) of the cell and *F* is Faraday's constant. The extrusion of Ca^2+^ by the pump is modelled by the following linear equation (for example see [25]):

(3b) $$j_{\text{out},\text{app}}=-P_{\text{out},\text{app}}{\left[\text{C}{\text{a}}^{2+}\right]}_{i}\text{,}$$

where *P*_out,app_ denotes the apparent extrusion permeability (m s^−1^) and relates to the real permeability as follows: *P*_out,app_ = *P*_out_/*κ*_tot_. The flux associated with sequestration was also assumed to be linear:

(3c) $$J_{\text{seq},\text{app}}=-k_{\text{seq},\text{app}}({\left[\text{C}{\text{a}}^{2+}\right]}_{i}-{\left[\text{C}{\text{a}}^{2+}\right]}_{\text{s}})\text{,}$$

where *k*_seq_,_app_ denotes the apparent sequestration rate constant (s^−1^) and relates to the real rate constant as follows: *k*_seq,out_ = *k*_seq_/*κ*_tot_·[Ca^2+^]_s_ is the steady state Ca^2+^ concentration. This approximate model is also known as a pool model [26]. Finally the basal influx *j*_basal,app_ was directly calculated from the steady state Ca^2+^ concentration and the extrusion parameter: *j*_basal,app_ = *P*_out,app_ [Ca^2+^]_s_.

### 2.8 Microelectrode model

In this study we used CASMs to measure the Ca^2+^ concentration after short depolarisations. If we assume equal activity coefficients in the Ringer and cytoplasm, the steady state cytoplasmic Ca^2+^ concentration can be directly estimated from the Nernst equation applied to the CASM potential:

(4) $$E_{\text{Ca}}=\frac{RT}{2F}\ln\left(\frac{{\left[\text{C}{\text{a}}^{2+}\right]}_{i}}{{\left[\text{C}{\text{a}}^{2+}\right]}_{\text{CASM}}}\right)+E^{0}\text{,}$$

where the steady state Nernst-potential is denoted by *E*_Ca_ (V), *R* is the gas constant and the temperature *T* was 293 K. The backfill Ca^2+^ concentration [Ca^2+^]_CASM_ in the microelectrode was 1.0 mM. The reference potential *E*^0^ was manually adjusted prior to the experiment such that the measured potential was 0 mV in Ringer solution; the Ringer solution contained 7 mM Ca^2+^.

Were the resistance and capacitance of the CASMs to be infinitely small and *V*_Ca_ to follow the Nernst equation over the whole range it could be used directly to estimate the cytoplasmic Ca^2+^ concentration. *V*_Ca_ is indeed close to the Nernstian value at [Ca^2+^]_*i*_ over 100 nM [14]. Unfortunately the resistance of these microelectrodes is very high, which will introduce a delayed microelectrode response. To allow for this delay we used a RC filter to calculate the model microelectrode potential *V*_Ca_:

(5) $$\frac{\partial V_{\text{Ca}}}{\partial t}=\frac{E_{\text{Ca}}-V_{\text{Ca}}}{\tau_{\text{CASM}}}$$

The response time of the microelectrode is denoted by *τ*_CASM_ (s). We assume that it is constant and depends only weakly on the microelectrode potential and [Ca^2+^]_*i*_.

### 2.9 Integration and parameter fitting

Assuming a linear transport equation a rather complex analytical solution consisting of an infinite series of exponentials with different time constants can be obtained using standard methods [27]:

(6a) $${\left[\text{C}{\text{a}}^{2+}\right]}_{i}(r,t)=\frac{j_{\text{Ca},\text{app}}}{r}\sum_{n=0}^{\infty }A_{n}\tau_{n}\sin\left(2\alpha_{n}\frac{r}{d}\right)(1-e^{-t/\tau_{n}})\;0\leq t<t_{\text{Ca}}$$

(6b) $${\left[\text{C}{\text{a}}^{2+}\right]}_{i}(r,t)=\frac{j_{\text{Ca},\text{app}}}{r}\sum_{n=0}^{\infty }A_{n}\tau_{n}\sin\left(2\alpha_{n}\frac{r}{d}\right)(1-e^{-t/\tau_{n}})e^{-(t-t_{\text{Ca}})/\tau_{n}}\;t\geq t_{\text{Ca}}$$

The amplitudes *A*_*n*_ and the time constants *τ*_*n*_ are:

(7) $$A_{n}=2\frac{\alpha_{n}\;\sin(\alpha_{n})}{\alpha_{n}-\sin(\alpha_{n})\cos(\alpha_{n})}$$

(8) $$\tau_{n}={\left(4\alpha_{n}^{2}\frac{D_{\text{app}}}{d^{2}}+k_{\text{seq},\text{app}}\right)}^{-1}$$

The constants *α*_*n*_ are the positive roots of the following equation:

(9) $$\tau_{P}\alpha \;\cos(\alpha )+(\tau_{D}-\tau_{P})\;\sin(\alpha )$$

where the diffusion time constant *τ*_*D*_ = *d*^2^/(4*D*) and the extrusion time constant *τ*_*p*_ = *d*/(2*P*).

The slowest time constant determines the overall recovery time. When there is no sequestration this time constant depends on the cell diameter *d*, the extrusion rate constant and the diffusion coefficient in a complex way. However, when diffusion is much faster than extrusion (extrusion limited) the time constant is simply *τ*_0_ = *d*/(6*P*_out_) and vica versa when diffusion is much slower than extrusion (diffusion limited) the time constant is simply *τ*_0_ = *d*^2^/(4*πD*_app_).

To compare the model with the results obtained from the experiments, we solved the transport equation simultaneously with the differential equation for the microelectrode potential. Although the analytical solution is very useful for theoretical analysis and testing, it requires many terms to obtain an accurate solution and does not allow for non-linearity. Therefore, we used a numerical approach for solving both equations, allowing more flexibility. Time integration was done using the forward Euler scheme and diffusion was calculated using a finite volume scheme (see for example [22]). The value of surface-to-volume ratio *r*_sv_ in Eq. (2) using this finite volume scheme is simply the ratio of the cell surface *S* and the volume of the first compartment at the membrane. Both solutions were calculated using a Matlab 6.5 mex-routine written in C++.

In a typical experiment several Ca^2+^ transients were measured in succession at different depths. The relative distance was reconstructed from the micromanipulator movements that were all either 10 or 20 μm. The diameter of the cell was estimated from all of these successive steps. Unfortunately the absolute position (offset) of the transient measured closest to the membrane was not known exactly. We therefore allowed the depth as an adjustable parameter within the limits of ±10 μm. The transients measured closer to the middle were therefore more reliable. Furthermore, the response time of the electrode was estimated to be about 1.5–2.5 s and was allowed to vary within these limits. The remaining four unknown parameters in the transport equation, together with the response time, were obtained from an optimal fit. A simplex search algorithm was used to find this least square fit. To increase reliability of the parameter estimates, several transients measured at different depths were fitted simultaneously. Also, any slow baseline drift was removed and the first two seconds of the transients, that show an electrical artefact, were not used during the fitting process.

## 3 Results

### 3.1 Changes in measured calcium with depth of penetration

**Fig. 1:**
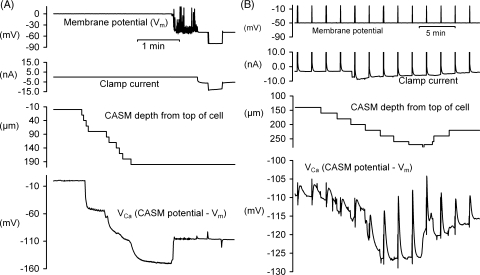
Parts of a representative experiment to record intracellular calcium with a Ca^2+^-sensitive microelectrode (CASM). (A) Start of experiment, showing sequence of microelectrode insertions into a snail neurone. The potential from the CASM (*V*_Ca_), which was referred to that from the membrane potential microelectrode, is shown on the bottom trace. The CASM was inserted first by making a series of 20 μm vertical downward movements starting with the electrode tip touching the top centre of the neuron. Once *V*_Ca_ had fallen below −130 mV (after reaching an apparent depth of 200 μm) a KCl-filled microelectrode was inserted to record the membrane potential (top trace), followed by a CsCl-filled microelectrode to pass clamp current (second trace, restricted to ±15 nA). The voltage-clamp was then activated and set to −50 mV. Finally a 30 mV hyperpolarisation for 20 s was applied to check that both the KCl microelectrode and CASM recorded the same change in potential. (B) Shows the same experiment 15 min later. Depolarisations (by 50 mV for 1 s) were applied every 2 min. After the first two depolarisations we moved the CASM deeper into the cell in steps of 10 or 20 μm to investigate how the responses changed. Once *V*_Ca_ started to increase rapidly we reversed the direction of movement. Parts of this figure are enlarged in Fig. 2.

At the start of all experiments the tip of a CASM was placed centrally on the top of the chosen neurone and moved down in 20 μm steps until there was a sudden fall in the potential, as shown in Fig. 1A. Inside the cell the CASM registers both the membrane potential (*V*_m_) and the change in *V*_Ca_ (the voltage, set to zero outside the cell, proportional to pCa). In this representative example the CASM was moved down in four steps before its potential changed from zero to about −50 mV. During a further six steps downward the CASM potential gradually became more negative, eventually more negative than −150 mV. By this time the electrode tip was 200 μm from the top of the cell, which had a diameter of about 240 μm. We then inserted the membrane potential microelectrode (the output of which was electrically subtracted from the CASM potential to give *V*_Ca_) and clamp current microelectrode, using manually-operated micromanipulators. To test that the CASM and *V*_m_ electrodes were recording the same change in *V*_m_, we then hyperpolarised the cell by 30 mV for 20 s. There were only small transient changes in *V*_Ca_, generated by electrical pickup and imperfect subtraction of rapid changes. We then (not shown in Fig. 1A) started to depolarize the cell by 50 mV for 1 s, repeated every 2 min, to allow an influx of Ca^2+^ ions. The recorded increase in [Ca^2+^]_*i*_ was very small; *V*_Ca_ increased from −107.5 to −106.4 mV, and then recovered. After the first depolarisation we raised the CASM by 60 μm and left it unmoved for 15 min to allow recovery from the initial penetrations.

As shown in detail in Fig. 1B, we then moved the CASM down every 2 min, between the 9th and 16th depolarisations, in six steps of 20 μm and two steps of 10 μm. (The third of these steps made the clamp current increase from −3 to −7 nA, but this was not correlated with any change in [Ca^2+^]_*i*_.) As the CASM tip was moved down the transient increases in *V*_Ca_ after each depolarisation became larger, and the steady-state *V*_Ca_ after each recovery became more negative. After the sixth step *V*_Ca_ had fallen to −127 mV, although after the next two it had risen slightly to −126 mV. After the last step down *V*_Ca_ rose to −118 mV before any depolarisation, suggesting the CASM had gone too far; although there was no change in the clamp current. We therefore then withdrew the CASM in several steps—two each of 10 and 20 μm. After the first 20 μm step up, the *V*_Ca_ paradoxically decreased, as if the upward movement somehow allowed a better seal. Such paradoxical improvements on withdrawal were often seen in the early part of an experiment.

**Fig. 2:**
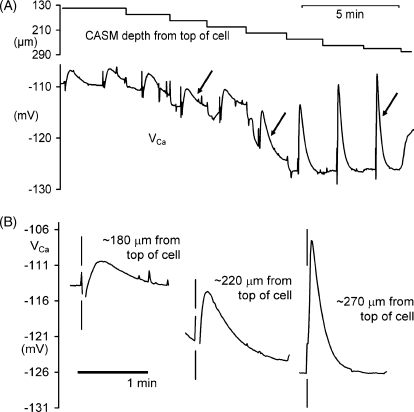
CASM movements up to the far side of the cell. (A) Enlargement of parts of the records from Fig. 1, showing eight separate microelectrode movements down into a snail neurone. After the first two depolarsations we moved the CASM down in six steps of 20 μm and two steps of 10 μm, as shown in the upper trace, to investigate how the responses changed. Each depolarisation was by 50 mV from the holding potential of −50 mV, for 1 s every 2 min. (B) Enlargements of the three *V*_Ca_ transients arrowed in part (A). The vertical lines indicate the times at which the membrane was depolarised by 50 mV for 1 s.

**Fig. 3:**
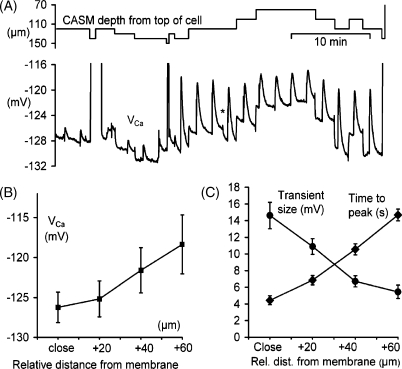
(A) An experiment showing *V*_Ca_ transients in response to depolarisations by 50 mV recorded at different depths near the far side of a cell (*V*_m_ and clamp current records not shown). The first six depolarisations were for 100 ms, the rest were for 1 s. The large excursions in the *V*_Ca_ trace occurred when the CASM tip appeared to reach the far side. Halfway through the trace, shown by (*), there was a spontaneous 2 mV change. (B) Data from six similar experiments showing steady-state calcium levels (as *V*_Ca_) plotted against the estimated distance of the CASM tip from the bottom of the cell. (C) Times to peak and sizes of *V*_Ca_ transients from six cells also plotted against distance from the bottom of the cell. *V*_Ca_ Data shown as means ± S.E.M.

Fig. 2 shows the *V*_Ca_ responses to the first nine depolarisations of Fig. 1B in more detail. As the CASM tip was moved closer to the far side of the cell the responses to the depolarisations became larger and faster. Presumably this reflects the fact that during the depolarisations Ca^2+^ entered the cytoplasm only at the cell membrane. Once *V*_m_ is returned to −50 mV, entry ceased and the Ca^2+^ both diffused into the cell interior and was pumped out by the plasma membrane Ca ATPase, or PMCA. In some experiments we were able to make several different sequences of downward and upward movements in the same cell to a depth at which, as shown in Fig. 3A, the apparent [Ca^2+^]_*i*_ rose rapidly. This experiment was done on a cell of diameter about 140 μm, and the depths at which the apparent [Ca^2+^]_*i*_ began to increase were, respectively, 140, 150 and 140 μm. In other words, it appears that when the CASM tip reached the far side of the cell it suffered a loss of sensitivity or caused a local leak. We could see no change in the clamp current associated with the sudden increases in *V*_Ca_. In many other cells, once the CASM created a sudden rise in [Ca^2+^]_*i*_ it took many minutes to recover, if at all.

The simplest explanation of the decreases in apparent [Ca^2+^]_*i*_ as the CASM was advanced deeper and deeper into a cell is that the electrode insertion induced a leak at the site of penetration. As the CASM was pushed in, its tip moved further away from the leak, which itself may be reduced by better sealing. During long experiments, as shown below, CASM movement had less dramatic effects on steady-state levels, suggesting that the leak declined with time.

In Fig. 3B we show mean steady-state *V*_Ca_ values against the estimated distance of the CASM tip from the bottom of the cell, from the early part of a total of six experiments in which the CASM was moved down in steps, like those of Figs. 1 and 3A. The bottom of the cell was taken as the point at which *V*_Ca_ rose rapidly without a change in membrane potential. Transients recorded within 20 μm of this were plotted as “close” to the membrane. The mean *V*_Ca_ close to the cell membrane was −126 mV, which corresponds to a pCa of 6.7; similar to the 6.76 (equivalent to a [Ca^2+^]_*i*_ of 170 nM) measured in snail neurones which had not been injected with buffers [13].

The *V*_Ca_ transients became faster as the CASM tips were moved closer to the far side of the cell. Fig. 3C shows the mean times to peak of the *V*_Ca_ increases from the same six experiments. From a best-fit line through the points, we can calculate that the mean rate at which the *V*_Ca_ peak moved towards the centre of the cell was 6 μm s^−1^. We also show in Fig. 3C the average size of the same transients, in mV from base to peak. The distance over which the transients decreased to half their (extrapolated) initial size of about 16 mV was about 45 μm, assuming that close to the membrane is equivalent to about 10 μm.

### 3.2 The effect of removing external calcium

**Fig. 4:**
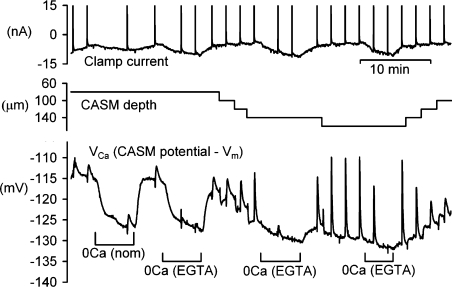
Representative experiment showing the effect of removing external Ca^2+^ on the [Ca^2+^]_*i*_ recorded by a CASM. The effects of nominally Ca-free solution applied when the CASM was 80 μm deep, and of Ca-free (EGTA) solution at three different depths were recorded.

If calcium ions, driven by the large electrochemical gradient, normally leak into the top of the cell around the tips of the microelectrodes, reducing external calcium should reduce the apparent [Ca^2+^]_*i*_. We have tested the effect of a possible leak by exposing cells to either nominally Ca-free solutions or the same with added EGTA. Measured with a CASM, the [Ca^2+^] of the nominal solution was about 20 μM, that of the EGTA Ca-free Ringer less than 10 nM. Fig. 4 shows a representative experiment in which the cell was superfused with the Ca-free solutions with the CASM at three different depths.

For the first two exposures to Ca-free solution the CASM was 80 μm deep in a cell of diameter about 170 mm. The Ca-free solutions caused *V*_Ca_ to decrease by 11 mV from, respectively, −115 and −116 mV. There was little difference between the effect of the two Ca-free solutions except that the EGTA solution almost abolished the second depolarisation-induced rise in Ca. As the CASM was moved deeper into the cell the steady-state *V*_Ca_ fell to −129 mV, and the reduction seen with Ca-free Ringer decreased to 4 and 3 mV, respectively. While some of the decreases seen with the removal of external Ca may reflect increased pump activity, it seems most likely that the elevated *V*_Ca_ levels seen near the middle of the cell are due to leakage round the microelectrodes where they cross the cell membrane.

### 3.3 The effect of CO_2_-bicarbonate buffering

We have done most of our experiments in HEPES-buffered Ringer, although it is known that CO_2_-bicarbonate-buffered solutions greatly increase intracellular pH buffering while reducing pH buffering power extracellularly [28,29]. It is possible that such a change in pH buffering might influence the diffusion of Ca^2+^ ions. We have therefore recorded the effects of changing the extracellular buffer from HEPES to CO_2_-bicarbonate on Ca^2+^ transients recorded at different depths. While it is well established [16,30] that the intracellular pH (pH_*i*_) decrease caused by CO_2_ entry leads to a decrease in steady-state [Ca^2+^]_*i*_, the Ca^2+^ transients were little changed (not shown). By 20 min after changing the superfusate to CO_2_ the Na-dependent chloride/bicarbonate exchanger [28] should have returned pH_*i*_ to normal, so we then again moved the CASM down in steps. This time the *V*_Ca_ baseline did not change, and the transient times to peak were very similar to those recorded in HEPES. In a total of four similar experiments, the average time to peak at distances of 0–20 μm was 4.20 ± 0.45 s in HEPES, and 4.35 ± 0.68 s in CO_2_-bicarbonate.

### 3.4 The role of calcium stores.

**Fig. 5:**
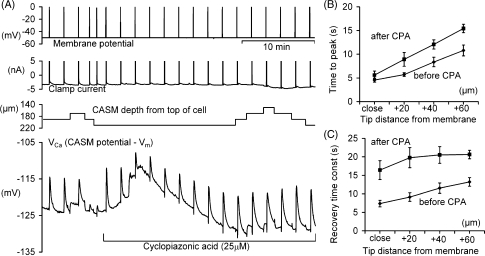
The effect of CPA on the calcium transients at different depths. (A) Representative experimental record. All depolarisations were by 50 mV for 1 s except for the fourth and fifth which were for 100 ms. After 8 min the perfusate was changed to one containing 25 μm CPA. The clamp current record was restricted to the range ±5 nA. (B) The effect of CPA on the mean times from the start of the depolarisations to the peak of the *V*_Ca_ transients before and after CPA are plotted for five different cells and at four different distances from the cell membrane. (C) The average time constants for the recovery of the depolarisation-induced *V*_Ca_ transients before and after CPA are plotted for the same transients as in (B).

It seems possible that the *V*_Ca_ gradients described above, and the higher steady-state apparent [Ca^2+^]_*i*_ than previously reported in cells which had added buffer, were partly due to mechanically-induced leakage from the endoplasmic reticulum calcium stores. Having penetrated the cell membrane the CASM must have been moved down through the endoplasmic reticulum and might have caused significant injury. We have therefore tested the effects of the SERCA pump inhibitor cyclopiazonic acid (CPA). It is well-established that CPA empties the Ca^2+^stores over a period of several minutes by blocking their uptake mechanism [31], and emptying the stores will presumably stop them leaking. Fig. 5 shows one of four similar experiments with CPA, in all of which (once the stores were empty) it had little effect on either the baseline *V*_Ca_ or the near-membrane *V*_Ca_ changes with CASM movement. The times-to-peak are trending to be later in CPA treated cells, although this was not very pronounced close to the membrane and statistically not significant (*p*-value assuming time-to-peak is later after CPA: 0.635 (*n* = 4)). For distances of 20, 40 and 60 μm from the membrane the times to peak were significantly longer (*p*-values assuming times-to-peak are later after CPA 0.952(5), 0.952(5) and 0.952(5)). The recovery time after the Ca^2+^ transients was clearly much longer in CPA treated cells (*p*-values assuming recovery times are longer after CPA: 0.984(4), 0.984(5), 0.972(5) and 0.996(5)), as shown in Fig. 5C. Interestingly the effect of CPA on recovery times was more pronounced for transients measured close to the membrane. Furthermore, the transient size showed a downward trend and decreased on average about 15% (nominal [Ca^2+^]_*i*_) in CPA treated cells. Although this percentage was statistically not significant (*p*-values assuming transients are smaller after CPA: 0.794(4), 0.845(5), 0.79(5) and 0.726(5)), it may suggest that a small fraction of the increase in [Ca^2+^]_*i*_ is caused by release of Ca^2+^.

### 3.5 The effect of BAPTA injection

**Fig. 6:**
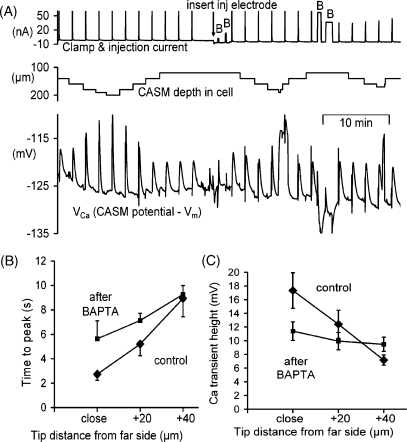
The effect of small and large BAPTA injections on Ca^2+^ transients at different depths. (A) Representative experiment. The cell was depolarised at 2 min intervals by 40 mV for 1 s. After 25 min a microelectrode filled with BAPTA was inserted at the point indicated by the arrow on the current record, and then BAPTA was injected four times where indicated by the letter B. The first two injections were estimated to load the cell to a concentration of 9 μm, the second two added an additional 125 μm. The clamp and injection current record was restricted to the range +60 to −10 nA. (B) The times to peak of Ca^2+^ transients recorded at three different distances from the cell membrane, before and after injecting BAPTA to a final concentration of 100–150 μm. (C) The *V*_Ca_ transient heights for the same three distances and BAPTA injections as in (A). Data from three cells.

Exogenous calcium buffers have been widely shown to influence Ca^2+^ signalling in a variety of cells; for example in nerve terminals [32] and Xenopus oocytes [33]. The fast buffer BAPTA is particularly interesting since it has similar properties to many of the indicators used to study Ca^2+^ signalling. We have therefore examined the effects of BAPTA, injected iontophoretically, on both the steady-state *V*_Ca_ and the Ca^2+^ transients induced by depolarisations. Fig. 6A shows a representative part of one of four similar experiments. Before the BAPTA injection we recorded *V*_Ca_ transients at several different CASM depths. At the closest approach to the membrane the time to peak was 2.9 s and *V*_Ca_ reached a peak of −107 mV. The first two BAPTA injections loaded the cell to give an estimated BAPTA concentration of 9 μM. At the closest approach to the membrane the *V*_Ca_ transient then had a time to peak of 6.6 s. After two more BAPTA injections giving an estimated final concentration of 135 μm, the transient time to peak at the closest point was again slower, at 9.1 s. In three of the experiments we were able to measure times to peak and the size of the *V*_Ca_ transients at three different depths before and after injecting BAPTA to a final concentration of 100–150 μm. The collected data are plotted in Fig. 6B and C. This shows that BAPTA tended to make the Ca^2+^ transients more uniform throughout the cell, both in times to peak and overall sizes.

Although the first two BAPTA injections had little effect on the steady-state *V*_Ca_, the second two lowered *V*_Ca_ from −128 to −131 mV. In a total of four similar experiments large BAPTA injections (raising the BAPTA concentration by over 100 μM) also lowered the steady state *V*_Ca_ by an average 4 ± 1.1 mV. This effect of BAPTA is similar to that reported before in snail neurones [14] in which intracellular CASM responses were calibrated by pressure-injecting BAPTA calibration solutions.

### 3.6 Computational modelling of Ca^2+^ transients in large snail neurones

In this section we present a minimal computational model that agrees fairly well with our experimental results. In the model we assume that during a 1 s current pulse (*I*_Ca,app_) Ca^2+^ enters the cell at the plasma membrane whence it diffuses into the interior with an apparent diffusion coefficient *D*_app_. Furthermore, Ca^2+^ is removed at the plasma membrane by a Ca^2+^ pump with extrusion permeability *P*_out,app_; we also allow for sequestration everywhere in the cell with rate *k*_seq_,_app_. Further mathematical details can be found in Section 2.

**Fig. 7:**
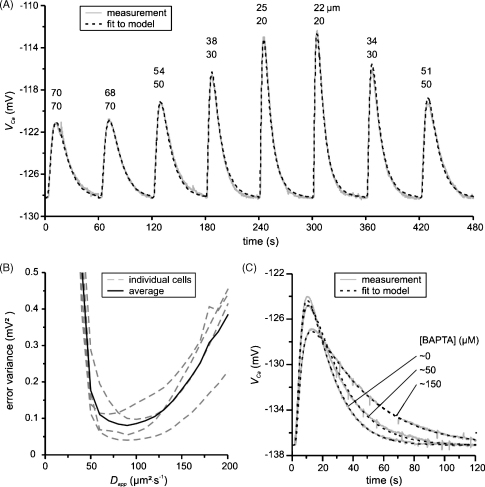
Results from the computational modelling of calcium transients. (A) Fitted Ca^2+^ transients measured at various depths; the cell diameter was 240 μm. The lower numbers above the peaks indicate the estimated distances from the membrane (from the micromanipulator) and the upper numbers are obtained from the optimal fit. The remaining apparent parameters are: *I*_Ca,app_ = −4.6 nA, *D*_app_ = 80 μm^2^ s^−1^, *P*_out,app_ = 11 μm s^−1^, *k*_seq_,_app_ = 0.047 s^−1^ and *τ*_CASM_ = 2.1 s. (B) Error variance of the fit as a function of the apparent diffusion coefficient. In four different cells least square fits were obtained with varying fixed apparent diffusion coefficients, all other parameters were allowed to change. In each cell we fitted at least three transients measured at distances ∼40, ∼60 and ∼80 μm. The mean error variance curve has a minimum error at 90 ± 20 μm^2^ s^−1^. (C) Transients measured with different BAPTA concentrations were simultaneously fitted to the model. The distances obtained from the fit were 52 μm (0 μM BAPTA), 54 μm (50 μM BAPTA) and 60 μm (150 μM BAPTA), respectively. *D*_app_ was calculated with Eq. (1) and all transport processes were scaled down with the total buffering power; the cell diameter was 240 μm. The parameters obtained from the fit were: *D*_e_ = 1000 μm^2^ s^−1^, *κ*_e_ = 65, *D*_b_ = 1830 μm^2^ s^−1^, *κ*_b_ = 26 (50 μM BAPTA), *κ*_b_ = 102 (150 μM BAPTA). The apparent diffusion coefficients at different BAPTA concentrations are: *D*_app_ = 102 μm^2^ s^−1^ (0 μM BAPTA), *D*_app_ = 125 μm^2^ s^−1^ (50 μM BAPTA) and *D*_app_ = 151 μm^2^ s^−1^ (150 μM BAPTA). The remaining real parameters (not scaled with *κ*_tot_*)* are: *I*_Ca_ = −63 nA, *P*_out_ = 120.0 μm s^−1^, *k*_seq_ = 2.9 s^−1^ and *τ*_CASM_ = 1.5 s.

In modelling Ca^2+^ transients at different depths we overcame our imprecise knowledge of the different parameters by estimating them using a least square fit. Fig. 7A shows eight simultaneously fitted transients that were measured in the middle of a long experiment at various depths ranging form close to the membrane at about 20 μm to about 70 μm away from the membrane. The depths estimated from micrometer movements are the lower values shown above the peaks. To fit the data these positions were allowed to vary within the limits of half a CASM step. The depths obtained from the fit (upper values) are reasonably close to the micrometer positions. Interestingly, modelled positions near the membrane are slightly shifted towards the centre of the cell, which might indicate lower mobility close to the membrane.

The optimal fit yielded an apparent diffusion coefficient of *D*_app_ = 80 μm^2^ s^−1^. Furthermore, the fit required an apparent sequestration rate constant of *k*_seq_,_app_ = 0.047 s^−1^, which corresponds to a time constant of ∼20 s. Attempts to fit the transients without sequestration failed.

The extrusion permeability was *P*_out,app_ = 11 μm s^−1^, which corresponds to a time constant of about 3.6 s, much shorter than the diffusional time constant of 18 s (see Section 2). This suggests that clearance is limited by diffusion rather than by the pump rate. In most cells we observed that after influx a rather steep gradient develops in the direction of the membrane, which fits the diffusion-limited case.

The response time was estimated to be *τ*_CASM_ = 2.1 s, which was slower than the 1 s that was measured in 10 μM Ca^2+^ Ringer. In most experiments the times-to-peak very close to the membrane ranged from 2.5 to 3.5 s. Because the time-to-peak near the membrane should equal the duration of the influx, the response time of the electrode in cytoplasm is about 1.5–2.5 s.

The estimated apparent diffusion coefficient for the recordings shown in Fig. 7 of *D*_app_ = 80 μm^2^ s^−1^ is higher than reported in other studies: ∼14 μm^2^ s^−1^ in frog muscle [34], ∼13 μm^2^ s^−1^ in cytoplasmic extracts of Xenopus oocytes [24], ∼20 μm^2^ s^−1^ in the axoplasm of metacerebral cells of *Aplysia californica* [23] and ∼10 μm^2^ s^−1^ in rod photoreceptor outer segments [35]. A diffusion coefficient of ∼10 μm^2^ s^−1^ has been reported in intact Myxicola axoplasm [36], but much higher values were seen in ATP-depleted axoplasm, and with high [Ca^2+^]_*i*_.

In most of these studies the diffusion coefficient was estimated using a simple model with only a few parameters. Our model contained more parameters that we varied to obtain a good fit. The parameters for influx, extrusion and sequestration and to some extent the positions and the response time may compromise the estimate of *D*_app_. We therefore calculated the mean error variance as a function of the apparent diffusion coefficient of four fits obtained from four different cells. While fixing the apparent diffusion coefficient, all other parameters were allowed to vary in order to obtain an optimal fit. In each cell we fitted at least three transients measured at distances ∼40, ∼60 and ∼80 μm and calculated the mean error variance as a function of *D*_app_*.* All cells gave a reasonable fit with a minimum error at 90 ± 20 μm^2^ s^−1^ (Fig. 7B). Since we allow all other parameters to compensate for an incorrect diffusion coefficient this value should be a conservative estimate. Attempts to fit the transients with *D*_app_ values smaller than 50 μm^2^ s^−1^ failed. Some cells fitted better than others, since the expected transient size did not always match perfectly when multiple transients were used. However, perfect fits could be obtained with any individual transient. Close inspection of the traces revealed that baseline variations could affect the transient size. Possibly the Ca^2+^ influx is reduced or removal mechanisms are enhanced at higher basal [Ca^2+^]_*i*_ levels. We still conclude that the diffusion coefficient is considerably higher (five to seven times) than reported for other cell types.

A large fraction of the cytoplasmic Ca^2+^ is bound to buffers. The properties and distribution of these Ca^2+^ buffers are therefore critical for Ca^2+^ mobility in the cytoplasm. Analysis of Ca^2+^ transients under differing free Ca^2+^ concentrations and exogenous Ca^2+^ buffering in snail neurones indeed showed that Ca^2+^ is quite well buffered in snail neurones [24]. To explain the high diffusion coefficient a large fraction of the Ca^2+^ buffers must be mobile. When we injected the cells with BAPTA, it clearly changed the *V*_Ca_ transients considerably, promoting spatially uniform global signals as described in Xenopus oocytes [33]. These were loaded with 48 μm Oregon Green to record [Ca^2+^]_*i*_, which may itself have influenced Ca^2+^ movement. Our Fig. 7C shows three transients measured at 50–60 μm from the membrane with different BAPTA concentrations; the nominal concentrations were 0, 50 and 150 μM. BAPTA slightly increased the times to peak (10.2, 10.6 and 12.6 s), reduced the size of the transients and slowed the recovery time significantly (13.6, 18.1 and 30.0 s). These values are consistent with a reduced clearance rate. Typically, if the Ca^2+^ mobility is not changed, reduction of the clearance rate slows the recovery time but also increases the time-to-peak. After normalisation of the times to peak we could only detect a slight reduction in the latency (<0.5 s) between 0 and 150 μM BAPTA, suggesting that the mobility of BAPTA is comparable to that of the endogenous Ca^2+^ buffers.

We fitted the three transients simultaneously to the model allowing a different exogenous Ca^2+^ binding ratio for each transient. The model gives a reasonable fit with an endogenous buffer diffusion coefficient of *D*_e_ = 100 μm^2^ s^−1^ and Ca^2+^ binding ratio of *κ*_e_ = 65. This suggests that the endogenous Ca^2+^-buffers have a comparable Ca^2+^ binding ratio to that in Aplysia axons [25] but appear to be much more mobile. The apparent diffusion coefficients for Ca^2+^ at the different BAPTA concentrations are: *D*_app_ = 102 μm^2^ s^−1^ (0 μM BAPTA), *D*_app_ = 12 μm^2^ s^−1^ (50 μM BAPTA) and *D*_app_ = 151 μm^2^ s^−1^ (150 μM BAPTA). The estimated diffusion coefficient of BAPTA was *D*_b_ = 183 μm^2^ s^−1^, which slightly increases the mobility of Ca^2+^ at higher BAPTA concentrations. Similar values for the BAPTA diffusion coefficient were reported by others [37–39].

## 4 Discussion

We have shown that with CASMs it is possible to record steady-state calcium levels and depolarisation-induced Ca^2+^ transients at different depths in large snail neurones. The decrease in calcium levels as the CASM was pushed deeper was probably due to increasing the distance from a site of leakage around the CASM and other microelectrode insertions at the top of the cell. We found that the sizes and kinetics of the Ca^2+^transients measured after brief (1 s) depolarisations to zero or −10 mV varied systematically and repeatably with distance from the plasma membrane at the bottom of the cell. Blocking the endoplasmic reticulum SERCA pump with CPA had little effect on the transient sizes and times to peak, but significantly slowed subsequent recovery times. Injecting BAPTA both reduced and slowed the transients close to the membrane while magnifying them deep inside the cell. By fitting our results to a mathematical model we calculate that the apparent Ca^2+^ diffusion coefficient is about 90 μm^2^ s^−1^.

### 4.1 The problem of leakage

Ion-sensitive and other non-patch microelectrodes inevitably make a hole in the cell membrane, which allows leakage in or out of the cell. Given the gradients, the ions whose leakage is most likely to distort the measured intracellular level are Na^+^ and Ca^2+^. Thus, Vaughan-Jones [40] recorded [Na^+^]_*i*_ in crab muscle with a Na^+^-sensitive microelectrode (NASM) inserted radially (perpendicular to the long axis) and found that low external Na caused a surprisingly rapid fall in [Na^+^]_*i*_. He considered that this might have been caused by the low external Na reducing a leak. This was later confirmed by experiments in which it was shown that NASMs inserted along the long axis into a cannulated fibre recorded only small changes in [Na^+^]_*i*_ when external Na was reduced, but when inserted radially the recorded [Na^+^]_*i*_ was at first high but fell as the NASM was pushed deeper [12].

Perhaps because the gradient is small and pH_*i*_ very well buffered no one has considered that pH-sensitive microelectrode measurements have been contaminated by leakage. On the other hand, [Ca^2+^]_*i*_ is very low and only moderately buffered. In our hands CASMs unfortunately do not perform well unless their tips are at least 1 μm in diameter [15,17]. Furthermore, only after much manipulation did CASMs record a low [Ca^2+^]_*i*_ which increased on depolarization. The initially high levels were recorded even in cells loaded with Fura-2 whose fluorescence indicated a normal low [Ca^2+^]_*i*_. Experiments in which CASMs were calibrated while inside neurones by injecting buffers led to the conclusion that the true [Ca^2+^]_*i*_ in quiescent voltage clamped snail neurones with injected buffer was 40 nM (equivalent to a CASM *V*_Ca_ of about −140 mV) and that CASMs usually cause a leak at the point of insertion [14].

The early experiments with CASMs were all done with a manually-operated micromanipulator, so that systematic movements were not attempted once a stable *V*_Ca_ was obtained. The current results were obtained with a motor-driven and remote-controlled micromanipulator. Typically, the CASM was moved down in several 20 μm steps before its potential changed at all. This shows that the cell membrane is very extensible, since the cell as a whole did not visibly move as the CASM was inserted. For example in Fig. 1A, the CASM was moved down by 80 μm before the potential first fell. As the CASM tip was then moved deeper the potential became more and more negative. This was probably due to a combination of movement away from the site of leakage, and a reduction in the size of the leak. It is hard to distinguish between these two possibilities, but the reproducible effects of movement as shown for example in Fig. 3A suggest that the leak was relatively constant, although tending to reduce with time. In some experiments the steady-state *V*_Ca_ appeared to stabilize some distance from the apparent far side of the cell, as shown in the control part of Fig 6A, and was not reduced further as the tip approached the far side of the cell. Presumably the leakage was by then too small to have a significant effect.

### 4.2 Ca^2+^ influx rather than Ca^2+^ release determines the increase in [Ca^2+^]_*i*_

Our finding that the peak of the *V*_Ca_ transient diffuses away from the membrane at a speed of about 6 μm s^−1^ confirms the long-held view that Ca^2+^ signalling which relies purely on diffusion will inevitably have a slow response time. The lack of effect of CPA, and the decline of the signal size with distance, rather unexpectedly both rule out significant regeneration of the signal as it spreads inwards. This is surprising, since snail neurones respond well to caffeine and certainly have the capacity for Ca-induced Ca^2+^ release (CICR) [41,16]. For example, the addition of a low concentration of caffeine slightly enhanced Ca^2+^ transients [41], while the removal of caffeine temporarily reduced the peak amplitudes suggesting that the stores were at least partly emptied [16].

Either the signal is large enough without any store contribution, or something about our experimental conditions has inhibited CICR. We depolarised the cell for 1 s to ensure a large Ca^2+^ load every 2 min, but this may have in some way have inhibited store release. On the other hand in other experiments we have avoided depolarising the cell for long periods, and not found that the first subsequent response was different. The distance over which the *V*_Ca_ transients fell to half their extrapolated initial size was about 45 μm, similar to the distance between the cell membrane and the nucleus. In normal-sized neurones of course the nucleus would be much closer.

We conclude that the main mechanism in our experiments that leads to an increase in [Ca^2+^]_*i*_ is transmembrane Ca^2+^ influx through voltage-gated Ca^2+^ channels opened by depolarisation and not massive release triggered by a small Ca^2+^ influx as in vertebrate cardiac muscle. The only previous direct measurements of Ca^2+^ movement in nerve cells without added buffers were described by Gorman et al. [42]. They were able to detect a rise in [Ca^2+^]_*i*_ only within about 40 μm of the membrane in Aplysia neurones after a 5 s depolarisation. Their CASMs were made with a different sensor (ETH 1001) to that which we used, and appear to have been relatively insensitive at low values of [Ca^2+^]_*i*_.

### 4.3 The transmembrane pump and sequestration by cytoplasmic organelles determine Ca^2+^ clearance

The depolarisation-induced Ca^2+^ influx is presumably cleared by extrusion across the cell membrane and sequestration to cytoplasmic organelles. The PMCA or Ca^2+^:H^+^ ATPase in snail neurones appears to exchange two extracellular protons for one intracellular Ca^2+^ at the expense of ATP [43]. When it is blocked by ortho-vanadate, the [Ca^2+^]_*i*_ does not fully recover from an influx but ends in a plateau. This suggests that the PMCA is the essential removal mechanism, since the endoplasmic-reticulum and mitochondrial uptake mechanisms are not inhibited by cytoplasmic *o*-vanadate [16].

Nevertheless, for many different cell types it has been shown that mitochondria participate in Ca^2+^ clearance when Ca^2+^ concentrations are high [44,45]. It is very difficult to measure the action of mitochondria in snail neurones directly. However, an increased muffling power at high Ca^2+^ concentrations [24] suggests that mitochondria may play a role close to the membrane where high transients occur.

Instead of reinforcing the Ca^2+^ signals, and thereby generating a fast travelling Ca^2+^ wave into the cell, the stores clearly play a role in the recovery of the Ca^2+^ transients. CPA significantly increased the Ca^2+^ clearance time from ∼13 to ∼18 s; these were measured in the centre of the cell and were corrected for the CASM response time. From this we estimate that the stores contribute about 25% to the clearance. This is lower but comparable to the result found in Purkinje cell somata from rat cerebellar slices, where blocking of the endoplasmic Ca^2+^ pump also significantly slowed the recovery time after influx [46].

### 4.4 Calcium transients change systematically with depth

Although the steady-state *V*_Ca_ levels were probably elevated by continuous Ca^2+^ entry, the recorded transients and the way they changed with depth were, we believe, physiologically significant. It seems unlikely that the kinetics of the diffusion of Ca^2+^ from the far side of the cell towards the centre would be changed by a constant or slowly-declining leak. Our average value for the speed at which a depolarisation-induced Ca^2+^ transient flows from the periphery to the centre of a nerve cell with no added mobile buffer was ∼6 μm s^−1^. We can find no previous measurements of this parameter in excitable cells, although several groups have modelled the process; (for example [8,12]). The only real measurement we have found was done with 100 μm Fluo-3 in an isolated atrial myocyte with inhibited sarcoplasmic reticulum Ca^2+^ pumps [10]. Our estimate from their Fig. 1 is that the calcium peak moved at least 10 times faster than in our observations, but the authors’ model showed that this was probably due to the Fluo-3. Their modelled data as shown in their Fig. 4 suggest that with no added mobile buffer their Ca^2+^ transients might travel to the centre of their model myocyte at a similar speed to that which we measure, although their time axis does not extend far enough. Furthermore, when the speed at which Ca^2+^ transients travel depends on diffusion, the velocity is not constant but varies with distance; typically velocity first increases and then decreases when the centre of the cell is approached. In contrast, true waves are constantly regenerated and tend to move with a constant speed [47].

### 4.5 Calcium diffusion in large snail neurones without added buffer

**Fig. 8:**
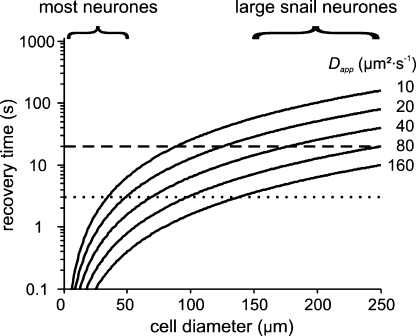
Calculated recovery times after a Ca^2+^ calcium transient for various diffusion coefficients as function of the cell diameter. Modelled calcium recovery times for spherical neurones of diameters up to 250 μm, assuming that diffusion is the rate-limiting process. Most neurones are in the range of 0–50 μm and recover in a few seconds; slow diffusion is not critical in those cases. Large snail neurones recover in about 10–20 s, which requires faster diffusion.

If recovery from a brief Ca^2+^ influx relies on extrusion at the plasma membrane, diffusion will become important for large cells because Ca^2+^ has to diffuse to the plasma membrane to be extruded. Moreover, the fastest possible clearance without sequestration is the diffusion limit. When the extrusion rate is much faster than diffusion, the recovery time constant is proportional to the square of the cell's diameter and inversely proportional to the diffusion coefficient (see Section 2). Because large snail neurones mainly rely on transmembrane extrusion mechanisms and do not seem to reinforce Ca^2+^-signals, the diffusion coefficient becomes a critical parameter for Ca^2+^ clearance and spatial signalling. Fig. 8 shows the recovery times, assuming that diffusion is the rate-limiting process, for various different diffusion coefficients as function of the cell's diameter. The recovery time of large snail neurones is about 10–20 s (dashed line), which requires a diffusion coefficient between 60 and 120 μm^2^ s^−1^. However, most snail and other animal nerve cell bodies are much smaller, with diameters between 5 and 50 μm. The recovery times of these cells is typically in the order of seconds (dotted line). Hence, with diffusion coefficients <20 μm^2^ s^−1^ the cell can still be cleared readily by the pump.

### 4.6 Why is Ca^2+^ more mobile in large snail neurones?

It is possible that large cells, that do not use Ca^2+^release mechanisms for signalling and rely on extrusion at the membrane for reasonably fast recovery, require a high Ca^2+^ mobility to function. A high mobility will also ensure fast signal transmission to the nucleus. This is quite different from large cells that use release mechanisms to trigger rapid on/off responses such as muscle, oocytes and chromaffin cells. These cells typically have low Ca^2+^ mobilities and do not rely as much on extrusion, but recycle internally released Ca^2+^. Small neurones and axonal regions with high surface to volume ratios represent another class of geometries, which do not require high Ca^2+^ mobility for fast recovery.

Because Ca^2+^ is well buffered with an estimated binding ratio of 65, the mobility of the endogenous mobile buffers must be high to explain the high apparent mobility of Ca^2+^. Possibly the ratio of mobile buffers to immobile buffers is higher in large snail neurones, or the diffusion coefficient of the mobile buffer fraction is much higher than in other cells. This may be related to less cytoplasmic crowding, for example by reduced cytoskeleton, causing the movement of proteins to be less constricted [48]. Axonal regions and muscle cells contain high concentrations of cytoskeletal components that may restrict or slow considerably the movement of larger calcium binding proteins compared to large snail neurones.

The great majority of studies on intracellular Ca^2+^ signalling have been done with indicators such as Fura 2, which can be used on all sizes and types of cell. But the interaction of the Ca^2+^ signals with the indicator may have subtle effects, which are hard to predict. It is to be regretted that CASMs so far have such large tips and slow responses that they can be used only on exceptionally large cells.
